# Low-dose-rate radiation exposure leads to testicular damage with decreases in DNMT1 and HDAC1 in the murine testis

**DOI:** 10.1093/jrr/rrt090

**Published:** 2013-09-11

**Authors:** Eun Ji Gong, In Sik Shin, Tae Gen Son, Kwangmo Yang, Kyu Heo, Joong Sun KIM

**Affiliations:** 1 Research Center, Dongnam Institute of Radiological and Medical Sciences (DIRAMS), 40 Jwadong-gil, Gijang-gun, Busan 619-953, Republic of Korea; 2 Natural Medicine Research Center, Korea Research Institute of Bioscience and Biotechnology, 30 Yeongudanji-ro, Cheongwon-gun, Chungbuk 363-883, Republic of Korea

**Keywords:** radiation effects, low-dose-rate radiation, testis, sperm

## Abstract

This study examined the effects of continuous low-dose-rate radiation exposure (3.49 mGy/h) of gamma rays on mice testicles. C57BL/6 mice were divided into sham and radiation groups (*n* = 8 each), and were exposed to either sham irradiation or 2 Gy for 21 days, 0.2 Gy for 2 days, or 0.02 Gy for 6 h of low-dose-rate irradiation. Testicular weight, seminiferous tubular diameter, and seminiferous epithelial depth were significantly decreased in the mice irradiated with 2 Gy at 1 and 9 days after exposure. Moreover, the low-dose-rate radiation exposure induced an increase in malondialdehyde levels, and a decrease in superoxide dismutase activity in the testis of mice irradiated with 2 Gy at 1 and 9 days after exposure. The sperm count and motility in the epididymis also decreased in mice irradiated with 2 Gy at 1 and 9 days after exposure, whereas there was no significant effect on the proportion of abnormal sperm. The expressions of DNA methlytransferases-1 and histone deacetylases 1 in testes irradiated with 2 Gy were significantly decreased compared with the sham group. In conclusion, the damage exerted on the testes and epididymis largely depended on the total dose of low-dose-rate radiation.

## INTRODUCTION

The systemic effect of radiation increases in proportion to the dose amount and rate [1, 2]. High doses and high-dose-rate radiation have been shown to be detrimental, causing cell death [3, 4]. In contrast, low-dose (≤0.2 Gy) radiation has been reported to exert various beneficial effects in living organisms [5, 6]. However, these studies do not adequately account for the systemic responses of low-dose-rate radiation. In particular, the association between accumulated radiation dose and adverse effects has not been clearly elucidated.

As one of the most radiosensitive organs, the testis can be significantly functionally impaired by very low doses of radiation [7]. Side-effects from radiation exposure can reduce the quality of life and can be dose limiting, leading to potential treatment reduction for patients with testicular cancer. Irradiation has long been established as an iatrogenic male reproductive toxin, as it can affect normal cells, especially rapidly proliferating ones such as spermatogenic cells. The latter are particularly susceptible to radiation-induced injury, and infertility is a common post-irradiation problem [8]. Ionizing radiation disturbs normal metabolism, proliferation and differentiation, which may lead to mutagenesis, apoptosis, and necrosis of radiosensitive cells. Such adverse events in the testis result in several abnormalities in spermatogenesis, potentially resulting in temporary or permanent infertility. Abnormalities include low sperm counts, increased abnormal spermatozoa, and defective sperm function [9–11]. Spermatogonia are especially sensitive to irradiation; doses as low as 0.1 Gy may damage these cells [8]. However, studies on mice suggest that low-dose-rate radiation does not damage stem spermatogonia and probably stimulates repair in damaged spermatogonial stem cells [12, 13]. Since guidelines regarding low-dose-rate radiation have yet to be established, there are no systematic protocols followed in studies examining these types of radiation.

Recent studies have investigated low dose (≤0.2 Gy) and low-dose-rate (≤6 mGy/h) radiation according to the recommendations of the United Nations Scientific Committee on the Effects of Atomic Radiation (UNSCEAR) [14]. Our previous study showed that low-dose-rate radiation exposure (3.49 mGy/h) did not cause adverse effects in BALB/c mice at dose levels of ≤2 Gy, whereas the testis weight decreased at a dose of 2 Gy [15]. In this study, we looked at the effects of irradiation at the same low dose-rate (3.49 mGy/h) in the testes of C57BL/6 mice.

## MATERIALS AND METHODS

### Animals and experimental procedures

Eight-week-old C57BL/6 male mice were obtained from the Central Lab. Animal Inc. (Seoul, Korea). These animals were kept in a specific pathogen-free facility and maintained at a temperature of 23°C ± 2°C, relative humidity of 50% ± 5%, artificial lighting from 08:00–20:00, and 13–18 air changes every hour. The mice were fed a standard animal diet. All animal experiments followed a protocol approved by the Institutional Animal Care and Use Committee of the Dongnam Institute of Radiological and Medical Sciences (DIRAMS).

The mice were randomly divided into four groups (*n* = 16 per group): sham, 0.02, 0.2 and 2 Gy radiation exposure. Low-dose-rate radiation (3.49 mGy/h) was administered in the long-term low-dose-rate radiation facility at the DIRAMS. Radiation exposure was conducted in a specific pathogen-free conditioned irradiation room equipped with a ^137^Cs source (185 GBq). Mice were placed in a cage on shelves located 2 m from the source providing sham, 2 Gy for 21 days, 0.2 Gy for 2 days, or 0.02 Gy for 6 h (3.49 mGy/h) exposure. Radiation exposure was continued for almost 24 h a day with the exception of 2 h a week during which the room was cleaned, bedding was changed, and the food and water was refreshed. Sham control mice for both treatment groups were placed on shelves in the same facility and shielded from the radiation.

To investigate the effects of radiation on the histopathological parameters of spermiogenesis, mice were sacrificed 1 and 9 days after irradiation, and the testes and caudal epididymis were immediately removed and weighed, taking into account that the duration of the cycle of the seminiferous epithelium in mice is 8–9 days [16].

### Evaluation of testis weight, seminiferous tubule characteristics and sperm characteristics

The right testes were fixed in Bouin's fluid solution for two days and embedded in paraffin according to routine procedures. Sections perpendicular to the long axis of the testes were made, and they were stained with hematoxylin and eosin. Testis sections from different treatments were used to measure epithelial height and diameter of the seminiferous tubules using an ocular micrometer. At least 50 tubular cross-sections were selected randomly from each section, with two sections taken from each animal, and examined under a microscope as previously described [10–11]. The left testes were flash-frozen and stored at − 70°C until required for biochemical analysis.

To measure sperm counts in the caudal epididymis and examine the frequency of sperm abnormalities, the right caudal epididymis was weighed and placed in 0.5 ml of saline and homogenized for 30 s. A 10-μl aliquot of sample was diluted with a solution of 0.25% trypan blue, 5% NaHCO_3_, and 0.35% formalin, and the sperm were counted in a hemocytometer. A part of each suspension was placed on a glass slide to make a smear, which was then air-dried and stained with 0.05% aqueous solution of eosin-Y. Deformed sperm, which were characterized by the lack of the usual hook (head appearing banana-shaped instead of curved), tail folded on the sperm body, bifid tail, and/or amorphic sperm (deshaped sperm head) were counted. At least 400 spermatozoa per group were observed at ×400 magnification. For viability assays, the left caudal epididymisshapen was removed, kept in 100 μl bovine serum albumin (5 mg/ml), and minced finely using small scissors. Release of sperm was permitted in the solution for 10 min at 37°C. Subsequently, sperm were collected with a micropipette and 20 μl was dispensed onto a slide for motility analysis. In each preparation, at least 100 sperm were counted in the same field. Live sperm were determined by counting all moving sperm. Sperm viability assessment was repeated in a new preparation from the same semen sample [10, 11].

### Assessment of superoxide dismutase activity and malondialdehyde level

Testes lysate was prepared in lysis buffer (150 mM NaCl, 20 mM Tris-HCl [pH 7.5], 1% NP40, 5 mM EDTA, 10 mM NaF, 1 mM Na_3_VO_4_, 1 mM DTT, and 1 × PIC) to evaluate oxidative stress following the protocol of superoxide dismutase **(**SOD) (STA-340, Cell Biolabs, Inc.) and MDA assay kit (Oxis International, Portland, OR, USA). The homogenates were centrifuged at 14 000 rpm for 30 min (4°C), and the pellet was then resuspended. The supernatant was used for the measurement of malondialdehyde (MDA) levels and SOD activity. The total protein level in the supernatants was determined by the Bradford method.

### Western blot analysis

The left testis tissue sonicates were solubilized in SDS-polyacrylamide gel electrophoresis sample buffer, and the protein concentration in each sample was determined using a Bio-Rad protein assay kit (Bio-Rad Laboratories, USA) with bovine serum albumin set as the standard. Total protein equivalents for each sample (70 µg protein per lane) were then separated in 10% SDS-polyacrylamide gels and electrophoretically transferred to an Immobilon-P^SQ^ transfer membrane (Roche Diagnositics, IN, USA). The membrane was immediately placed into a blocking solution (5% nonfat milk) at room temperature for 1 h. The membrane was incubated with a diluted primary antibody for DNA methlytransferases (DNMT)-1 (D4692, 1:1000; Sigma Aldrich. Inc., USA), histone deacetylases (HDAC)-1 histone deacetylation catalyzation (ab7028, 1:8000, Abcam, UK), and β-actin (1:10 000, Sigma Chemicals Co.) in TBS-T buffer (Tris-HCl based buffer with 0.2% Tween 20, pH 7.5) at 4°C overnight. After four washes with TBS-T for 10 min each, the membrane was incubated with the secondary antibody polyclonal anti-rabbit antibody or monoclonal anti-mouse antibody (1:10 000, Sigma Chemicals Co.) in TBS-T buffer at room temperature for 1 h. Horseradish-conjugated secondary antibody labeling was detected by enhanced chemiluminescence and exposure to a radiographic film. Pre-stained blue markers were used for molecular weight determination. The bands were quantified using Scion Image Beta 4.0.2 for Windows XP software (Scion, Frederick, ME, USA).

### Statistical analysis

The data are reported as means ± SEM. The data were analyzed using one-way analysis of variance (ANOVA) followed by a Student-Newman-Keuls *post hoc* test for multiple comparisons. Statistical significance was considered for *P* < 0.05.

## RESULTS

### Change of body and testis weight

**Fig. 1.**
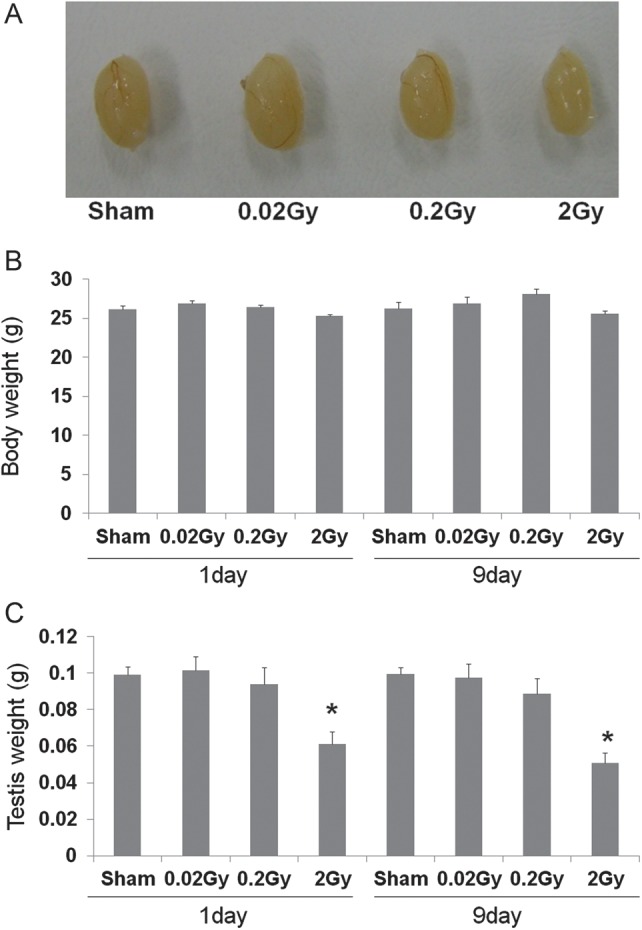
Effect of low-dose-rate radiation on body and testis weight at 1 and 9 days after irradiation. (**A**) Macroscopic view of the testis dissected from mice irradiated with sham, 0.02, 0.2 and 2 Gy low-dose-rate irradiation at one day after exposure. (**B**) No significant changes were noted in the body weight of mice between the irradiation and non-irradiation groups. (**C**) Although there was no significant difference in testis weight between the sham, 0.02 and 0.2 Gy irradiated groups, the exposure to 2 Gy of low-dose-rate radiation caused a significant decrease in testis weight at 1 and 9 days after exposure. Values are presented as mean ± SE for eight mice in each group. *Indicates significant difference at *P* < 0.05 vs the sham group.

Body weights were compared between the sham and low-dose-rate irradiated mice at 1 and 9 days after radiation exposure. There was no significant difference in body weight when exposed to 0.02, 0.2 or 2 Gy radiation (Fig. 1B). Further, there was no significant difference in testis weights between the sham, 0.02 and 0.2 Gy irradiated groups at both 1 and 9 days after radiation exposure. However, testis weight was significantly decreased in the 2 Gy irradiated group when compared with the sham group at both 1 and 9 days after radiation exposure (*P* < 0.05) (Fig. 1A and C).

### Seminiferous tubular diameter and epithelial depth

**Fig. 2.**
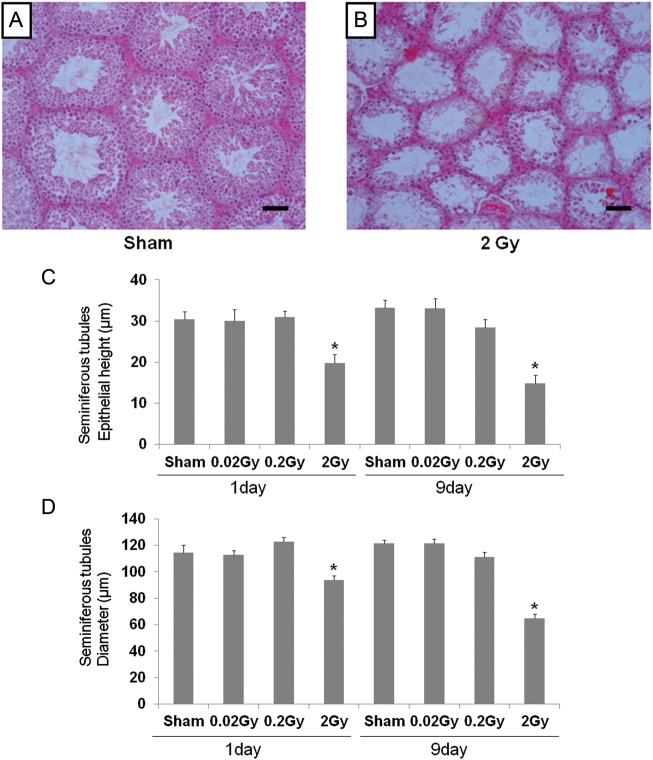
Histopathological testicular changes due to low-dose radiation at 1 and 9 days after irradiation. Representative images (H&E stain, ×200 magnification) showing histological changes in the testis, in the sham group (**A**) and in the 2 Gy irradiated group (**B**). Histological features of irradiated testis indicating a marked reduction in the number of spermatogenic cells, as well as a marked reduction in the diameter (**C**) and epithelial height (**D**) of seminiferous tubules. Values are presented as the mean ± SE of eight mice in each group. *Indicates significant difference at *P* < 0.05 vs the sham group. Scale bar = 30 µm.

To elucidate the toxicity of low-dose-rate radiation, testis sections were used to measure the epithelial height and diameter of the seminiferous tubules. The epithelial height of the seminiferous tubules was significantly decreased 1 and 9 days after irradiation with a dose of 2 Gy when compared with the control group (Fig. 2A, B and C). Furthermore, diameter of the seminiferous tubules was also significantly decreased 1 and 9 days after irradiation with a dose of 2 Gy when compared with the control group (Fig. 2A, B and D). There was no significant difference in epithelial height or tubular diameter in the 0.2 or 0.02 Gy irradiated groups compared with the sham group at both 1 and 9 days after radiation exposure (Fig. 2C and D).

### SOD activity and MDA level in the testis

**Fig. 3.**
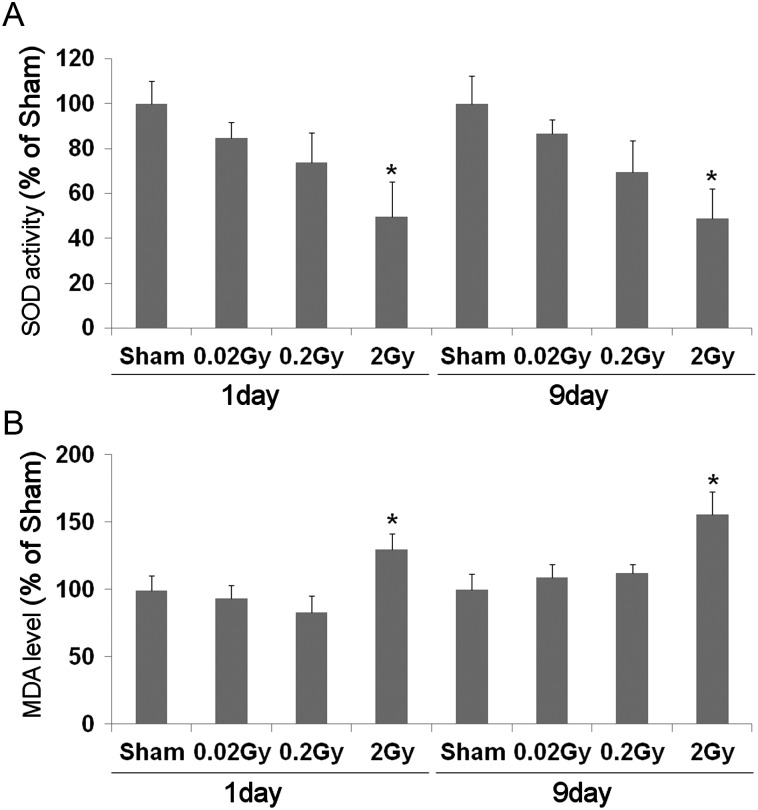
Effect of low-dose-rate radiation on testicular superoxide dismutase (SOD) concentration (**A**) and malondialdehyde (MDA) levels (**B**) at 1 and 9 days after irradiation. Values are presented as the mean ± SE of eight mice in each group. *Indicates significant difference at *P* < 0.05 vs the sham group.

SOD activity was illustrated in Fig. 3A. SOD activity of testis was significantly decreased in mice irradiated with 2 Gy of low-dose-rate irradiation at 1 and 9 days after irradiation; however, no statistically significant differences were observed in the 0.02 and 0.2 Gy groups (*P* < 0.05, Fig. 3A). The lipid peroxide level was measured according to the formation of MDA. The MDA level in the 2-Gy irradiated groups was remarkably higher than in the control group at 1 and 9 days after irradiation; however, no statistically significant differences were observed in the 0.02 or 0.2 Gy groups (*P* < 0.05, Fig. 3B).

### Sperm count, sperm motility and sperm abnormalities in the epididymis

**Fig. 4.**
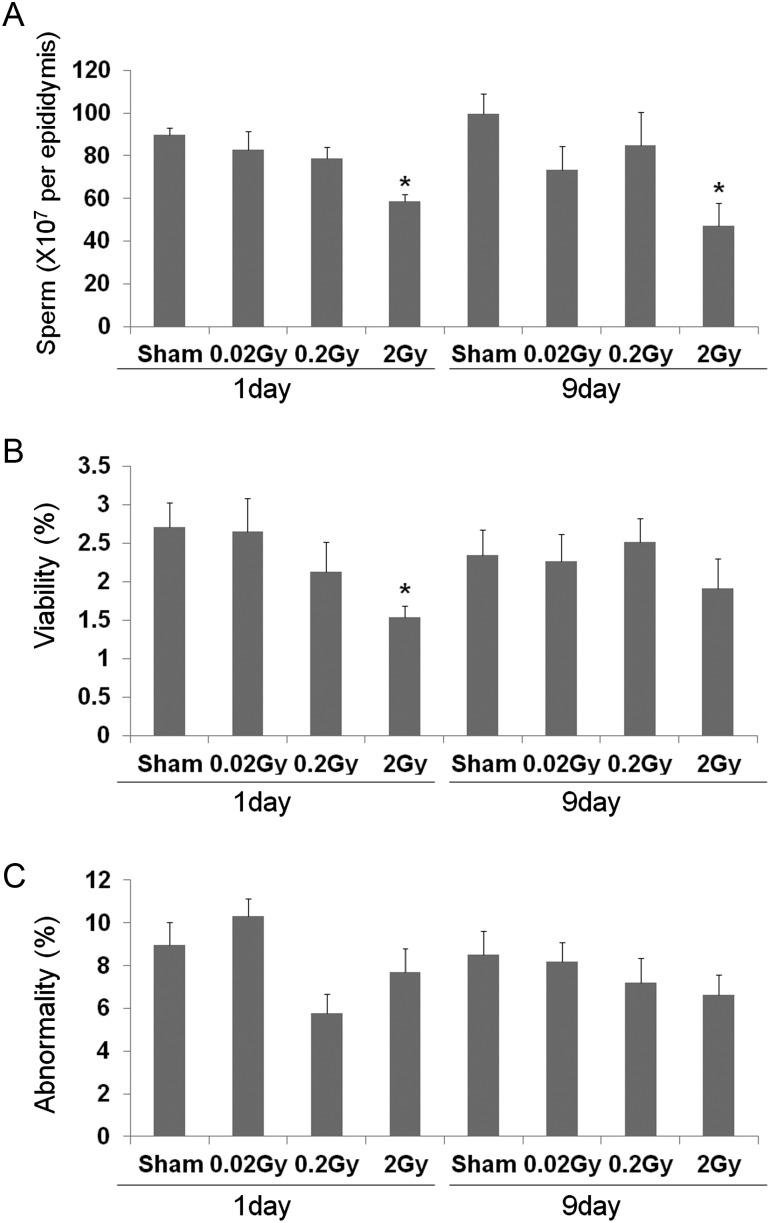
Effect of low-dose radiation on epididymal sperm characteristics at 1 and 9 days after irradiation. Sperm count (**A**), sperm motility (**B**), and sperm abnormalities (**C**) in the epididymis are observed. Values are presented as the mean ± SE of eight mice in each group. *Indicates significant difference at *P* < 0.05 vs the sham group.

The sperm count and motility in the epididymis were similar among the sham, 0.02 and 0.2 Gy irradiated groups at both 1 and 9 days after radiation exposure. However, there was a significant decrease in sperm motility and number of sperm in the caudal epididymis in the 2-Gy irradiated group compared with the sham group (*P* < 0.05, Fig. 4A and B). There was no significant change in the proportion of abnormal sperm in all the radiation exposure groups at either 1 or 9 days after radiation exposure. (Fig. 4C).

### Expression of DNMT1 and HDAC1 in the testis

**Fig. 5.**
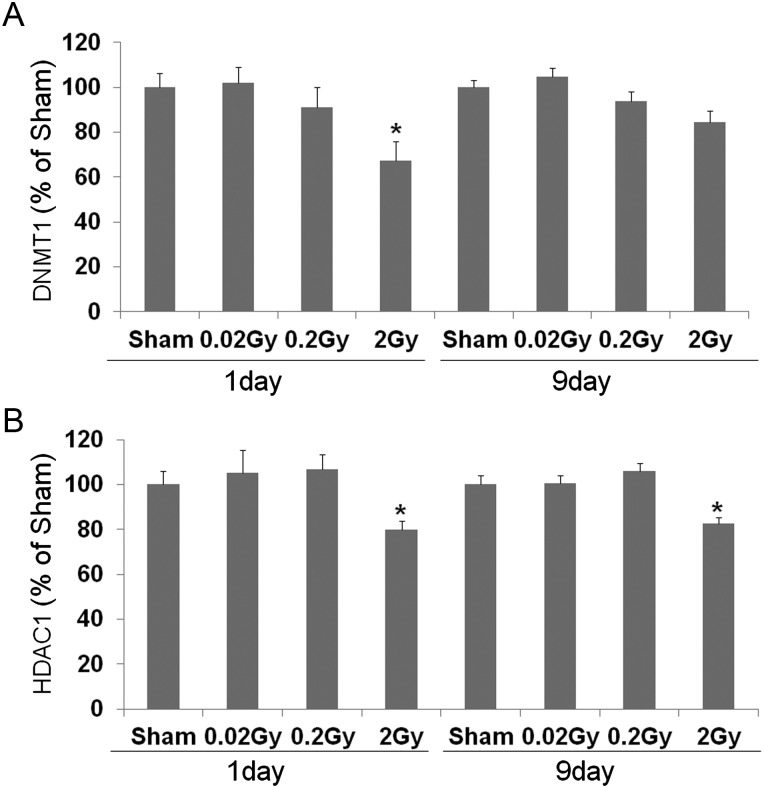
Effect of low-dose-rate radiation on testicular levels of DNMT1 (**A**) and HDAC1 (**B**) at 1 and 9 days after irradiation. The relative expression levels of DNMT1 (A) and HDAC1 (B) were determined through densitometric data analysis and normalized to the β-actin signal from five different samples (mean ± SE). *Indicates significant difference at *P* < 0.05 vs the sham group.

Western blot was used to determine the expression levels of DNMT1 and HDAC1. The relative expression levels of DNMT1 and HDAC1 were calculated after normalization to the β-actin bands from five different samples of irradiated mice testis (Fig. 5).

There were no further changes in DNMT1 expression levels detected at 0.02 or 0.2 Gy irradiation. In contrast, 2-Gy irradiation significantly decreased the DNMT1 expression level at 1 day after radiation exposure. At 9 days after radiation exposure, the DNMT1 expression levels were decreased; however, this decrease was not significant (*P* < 0.05, Fig. 5A).

A strong HDAC1 signal was detected for the sham, 0.02 and 0.2 Gy irradiated testis. The signal decreased significantly after 2 Gy irradiation at both 1 and 9 days after radiation exposure (*P* < 0.05, Fig. 5B).

## DISCUSSION

In the present study, we investigated the effects of chronic low-dose-rate (3.49 mGy/h) radiation on the testes in order to understand the underlying toxicity of low-dose-rate irradiation.

In a previous study, BALB/c mice that were exposed to a total dose of 0.02, 0.2 and 2 Gy were found to be healthy and did not show any significant changes in body weight or peripheral blood components [15]. However, the mice irradiated with a dose of 2 Gy had significantly decreased testis weight [15]. Although the mouse strain used in the present study differed from that used in the previous study, we noted that the C57BL/6 mice also exhibited a significant decrease in testis weight. Further, histological studies and sperm evaluation also demonstrated changes consistent with the findings of decreased testis weight. It is well known that high dose-rate irradiation adversely affects cell proliferation, and has caused death and depletion of spermatogonia cells and their subsequent generations, thereby negatively impacting testis weight [10, 11, 17]. In the previous study, testis weight declined in a dose-dependent manner after radiation exposure, but this did not depend on the dose rates [18]. Moreno *et al.* reported that when gonocytes were irradiated during their cell cycle arrest, there was no variation in the response observed for dose rates from 0.6–166.6 mGy/min [18]. The strong effect of radiation on germ cells is evident even at very low dose-rates, with the appearance of clusters of apoptotic gonocytes [19]. This observation may be explained by the homogeneous and continuous block of all gonocytes during the entire duration of radiation exposure. This study examined the long-term effects of low-dose-rate radiation, and indicated a decrease in testis weight and increase in the proportion of abnormal tubules. Most radiation damage arises in biological systems due to ROS generation. Exposure to radiation induces DNA damage, cell death, and severe impairment of various organs through the generation of ROS [20]. In the present study, we noted that the activity of SOD (a ROS scavenger) was decreased, whereas the level of MDA (a product of lipid peroxidation) was increased following exposure to low-dose-rate radiation.

Other studies have reported that low-dose-rate (0.7 mGy/h) radiation at a dose of 2 or 4 Gy does not affect spermatogonia [12, 13]. Those authors had assessed the toxicity of low-dose-rate radiation by evaluating sperm abnormality. In the present study, we also did not note a significant difference in sperm mutation, even at 2 Gy. The previous study suggested that low-dose-rate radiation activates a signal pathway related to DNA repair and/or apoptosis of abnormal cells, which then contributes to a decrease in abnormal sperm [12, 13]. However, in this study, other parameters related to spermatogenesis confirmed the presence of testicular injury in mice continuously exposed to 2 Gy of low-dose-rate radiation. In particular, there was a concurrent decrease in sperm counts after irradiation in the caudal epididymis.

DNMT1 and HDAC1 play important roles in gene expression during spermatogenesis [20]. Interestingly, in fertile patients found to have arrest of sperm maturation, the seminiferous tubules lack the DNMT1 protein [21]. As spermatogonia are mitotically active cells [22], expression of DNMT1 may be required to maintain the DNA methylation pattern during DNA synthesis [22]. In addition, DNMT1 has been found to have a direct role in restoring epigenetic information during the DNA repair processes, which may be mediated by its binding to the proliferation cell nuclear antigen binding domain [23]. Our results show a significant decrease in testicular DNMT1 after 2 Gy of low-dose-rate radiation. DNMT1 has been reported to bind to HDAC1 and function in the co-expression in the nuclei of spermatogonia [21, 24]. Histone hyperacetylation has been reported to play an important role in the production of fertile spermatozoa [25, 26]. In this study, HDAC1 was significantly decreased in the testis after 2 Gy of radiation exposure. The decrease in the expression levels of DNMT1 and HDAC1 in irradiated testis may be the part of the mechanism through which low-dose-rate irradiation results in testicular injury.

The duration of the cycle of the seminiferous epithelium in mice is 8–9 days [16]. Thus, based on the duration of the mouse spermatogenesis cycle, we assessed the mice at 1 and 9 days after irradiation exposure. As the spermatogonial stages are highly sensitive to irradiation, this may result in the progressive decline in sperm number and shrinkage of the seminiferous tubules. This process could account for the decrease in testis weight, which occurs over 9 days after high-dose-rate irradiation exposure in mice [10, 11]. In the present study, we chose 1 and 9 days after irradiation. The findings of the present study indicated that the low-dose-rate irradiation exposure at 2 Gy decreased the seminiferous tubule size and epithelial height, even 1 day after irradiation, and continued to decrease until 9 days after irradiation.

## CONCLUSION

In conclusion, despite a low-dose-rate radiation, our study found that when mice testis was irradiated with 2 Gy at a 3.49 mGy/h dose rate, there was significant testicular and sperm damage and decreased DNMT1 and HDAC1 expression.

## FUNDING

This research was supported by the National R&D program, through the Dongnam Institute of Radiological & Medical Sciences (DIRAMS), funded by the Ministry of Education Science and Technology, Korea (50496-2013).
